# Marginal adaptation of zirconium dioxide crowns prepared with four different finish lines: An *in vitro* study

**DOI:** 10.6026/973206300221569

**Published:** 2026-03-31

**Authors:** Aviraj Gill, Priyabrata Jena, Saumya Sharma, Sanjeev Singh, Avinash Kumar, Baby Jaman, Pratik Surana

**Affiliations:** 1 Department of Prosthodontics and Crown and Bridge, Maitri College of Dentistry and Research Centre, Anjora, Durg, Chhattisgarh, India; 2 Department of Pedodontics and Preventive Dentistry, Maitri College of Dentistry and Research Centre, Durg, Chhattisgarh, India

**Keywords:** Marginal integrity, finish line, zirconia crown

## Abstract

Poor marginal adaptation of zirconia crowns may lead to microleakage, secondary caries and reduced restoration longevity. Marginal integrity is therefore critical for the clinical success of CAD/CAM zirconia crowns. Therefore, it is of interest to evaluate the effect of four finish line designs-rounded shoulder, chamfer, bevelled shoulder, and sloping shoulder-on the marginal fit of zirconia crowns. The rounded shoulder showed superior marginal adaptation compared to chamfer, while bevelled shoulder showed no advantage over sloping shoulder. Rounded shoulder and chamfer finish lines may therefore be more favorable for CAD/CAM zirconia crowns.

## Background:

The long-term success of full coverage crown restorations is influenced by multiple clinical and material-related factors, among which marginal adaptation plays a critical role [1, 2]. Marginal fit may be affected by variables such as finish line configuration, repeated ceramic firing cycles, and the type of cementation material used. These aspects have been extensively investigated in both metal-ceramic and all-ceramic restorations [3 4-5]. Marginal adaptation refers to the interface between the cemented restoration and the prepared tooth surface. This area is particularly susceptible to plaque accumulation and recurrent caries due to dissolution of the luting agent and the presence of surface irregularities [6]. Improved adaptation of the restoration to the tooth structure reduces the likelihood of marginal leakage, secondary caries, and periodontal complications [7]. Conversely, rough and irregular marginal junctions increase the effective marginal length and compromise the overall fit of the restoration [8]. Retention and marginal discrepancy are therefore considered key determinants of the clinical longevity of complete crown restorations. Over the past two decades, increasing emphasis on esthetics and biocompatibility has driven the development of metal-free ceramic systems with enhanced mechanical properties [9]. This evolution has led to the widespread use of all-ceramic restorations, which are capable of meeting biomechanical demands while demonstrating clinical performance comparable to conventional metal-ceramic crowns [10]. Among the available ceramic materials, zirconium-based ceramics have gained significant attention due to their favorable combination of strength, toughness, and esthetic potential. Zirconium occupies a distinctive position among oxide ceramics and is widely used for crowns, fixed dental prostheses, and implant abutments across a range of clinical situations [11, 12]. Therefore, it is of interest to evaluate and compare the effect of different finish line designs-rounded shoulder, chamfer, beveled shoulder, and sloping shoulder-on the marginal fit of zirconia crowns.

## Materials and Methodology:

The present *in vitro* study was conducted in the Department of Prosthodontics and Crown and Bridge, Maitri College of Dentistry and Research Centre, Anjora, Durg, Chhattisgarh. A maxillary right molar typodont tooth was prepared to receive a zirconia crown following standard prosthodontic principles. The tooth preparation included a circumferential 1.0 mm chamfer finish line, with a 2.0 mm reduction on the functional cusp and a 1.5 mm reduction on the non-functional cusp. All preparations were carried out using medium-grit diamond burs to ensure standardized tooth reduction. Following preparation, the maxillary typodont was digitized using a 3Shape D700 laboratory scanner. The digital scan was utilized to replicate the acrylic typodont and fabricate a standardized master model, which served as a stable and reproducible reference for all subsequent impressions and measurements. Using this master model, zirconia crowns were fabricated through a completely digital workflow. The acquired scan data were exported as STL files and electronically transferred to 3M for data cleaning and processing. The processed files were then used by the dental laboratory employing Exocad (Germany) digital workflow software (Las Vegas, Nevada, USA) for digital articulation, virtual wax-up, and final crown design. The zirconia die was prepared digitally, and each crown was seated on the corresponding die without the use of cement or any intermediary material. Marginal gap measurements were obtained at eight predetermined locations-mesial, distal, buccal, palatal, and at the mesio-buccal, mesio-lingual, disto-buccal, and disto-lingual line angles-following the methodology described by Bindl and Mörmann [13]. The measurements represented the vertical component of the marginal gap and were recorded based on the definition of marginal fit proposed by Holmes *et al.* wherein absolute marginal discrepancy is defined as the distance between the crown margin and the finish line of the prepared tooth [14]. A total of thirty-two specimens were prepared in accordance with Shillingburg's principles, with specimens allocated equally among four groups based on finish line design. Group A consisted of shoulder finish line preparations, Group B chamfer finish line preparations, Group C beveled shoulder finish line preparations, and Group D sloped shoulder finish line preparations. Standardized tooth preparations were digitally generated using CAD software to fabricate resin dies for all groups. All specimens were evaluated under a scanning electron microscope (SEM) at 150x magnification, and representative images were obtained. Marginal gap measurements were recorded perpendicular to the crown margin to assess the vertical component of marginal discrepancy. Prior to SEM evaluation, all samples were sputter-coated with a thin layer of gold to serve as a conductive medium and facilitate accurate image acquisition.

## Results:

The present experimental study evaluated the marginal gap of CAD/CAM zirconia crowns fabricated with four different finish line designs using 32 samples, with five specimens per group (round shoulder, chamfer, beveled shoulder, and sloping shoulder) (Table 1 - see PDF). The round shoulder margin showed the lowest mean marginal gap (23.80 ± 0.94 µm), followed by chamfer (39.50 ± 3.29 µm), beveled shoulder (50.40 ± 0.78 µm), and sloping shoulder (62.74 ± 3.35 µm) designs. One-way ANOVA revealed a statistically significant difference in marginal gap values among the four groups (f = 233.2; p = 0.001) (Table 2 - see PDF). Intergroup comparison demonstrated that the round shoulder margin exhibited significantly lower marginal gaps compared to all other finish line designs, with statistically significant differences also observed between chamfer, beveled shoulder, and sloping shoulder groups (p = 0.001) (Table 3 - see PDF). Although statistically significant differences were noted, all margin designs demonstrated marginal gaps within clinically acceptable limits. Overall, preparations with a round shoulder finish line showed superior marginal adaptation compared to other margin designs. Although statistically significant differences were observed among all groups, the marginal gaps recorded for all finish line configurations were within clinically acceptable limits, and no clinically relevant marginal discrepancy was identified.

## Discussion:

Marginal integrity is a critical determinant of the clinical success and longevity of zirconia crown restorations [15]. Previous investigations on metal-ceramic systems have reported compromised marginal fit, primarily attributed to differences in thermal contraction between porcelain and metal, alloy composition, and finish line design. The advent of all-ceramic systems aimed to overcome these limitations by offering improved biocompatibility, enhanced strength, superior esthetics, and more predictable marginal adaptation. Among these materials, zirconia-based ceramics represent a significant advancement in restorative dentistry; however, evidence regarding the influence of finish line design on their marginal integrity remains limited [15, 16]. The findings of the present study revealed that zirconia crowns fabricated with a rounded shoulder finish line demonstrated significantly lower marginal discrepancy compared to those prepared with a chamfer finish line, leading to acceptance of the alternative hypothesis. These results are consistent with previous studies by Quintas *et al.* [5] and Cho *et al.* [17], who also reported superior marginal adaptation with shoulder-type preparations. In contrast, Komine *et al.* [18] observed reduced marginal discrepancies for shoulder preparations but did not identify statistically significant differences between the groups. Similarly, Suárez *et al.* [19] reported no significant variation in marginal adaptation among different finish line designs. It is noteworthy that these investigations employed mechanized specimen preparation rather than clinically prepared natural teeth, which may minimize variability but does not fully replicate clinical conditions. Although the modified shoulder finish line demonstrated lower marginal discrepancy values than the chamfer finish line in the present study, both designs consistently showed marginal gaps within the range of clinically acceptable limits (<120 µm). This suggests that while finish line design influences marginal integrity, both configurations may be considered clinically acceptable when proper preparation and fabrication protocols are followed. A 90-degree shoulder finish line with a rounded axiogingival line angle has been recommended for both all-ceramic and metal-ceramic restorations due to its resistance to distortion and favorable stress distribution.

The rheological behavior of zirconia framework materials differs from that of metal-ceramic systems, particularly with respect to creep and deformation characteristics. These differences may account for the increased marginal discrepancies observed in chamfer finish line designs in the present study. Additionally, shoulder-type preparations have been reported to offer greater resistance to distortion during fabrication and firing procedures. Previous studies evaluating In-Ceram crowns have shown no statistically significant differences in marginal fit between shoulders and chamfer finish line designs. However, differences in material properties and fabrication techniques may explain the variation in outcomes when zirconia-based restorations are evaluated [20]. In the present study, the beveled shoulder finish line exhibited better marginal integrity than the sloping shoulder design; however, both finish line configurations showed greater marginal discrepancies when compared with shoulder and chamfer preparations. These findings are consistent with those of Jalallian *et al.*, who reported improved marginal adaptation with shoulder bevel preparations in porcelain-fused-to-metal restorations [21]. Furthermore, Panno *et al.* [22] observed an average marginal gap of 45 µm for shoulder bevel designs, while Gavelis *et al.* and Faucher *et al.* reported mean marginal gap values of 44 µm and 62 µm, respectively [23,24]. Variations in marginal adaptation associated with different finish line designs highlight the importance of careful and standardized tooth preparation, as preparation geometry can influence the precision of CAD-CAM fabricated high-strength ceramic crowns such as zirconia [25, 26]. Finish line design plays an important role in determining the marginal adaptation of zirconium dioxide crowns. Variations in preparation geometry can influence the seating of the restoration and the precision of the marginal fit. In the present *in vitro* study, differences in marginal adaptation were observed among the evaluated finish line designs, indicating that preparation design may affect the final prosthetic outcome. Proper selection of finish line design may therefore contribute to improved marginal integrity and long-term clinical success of zirconia crowns [27, 28]. To date, there is a lack of studies comparing beveled shoulder and sloping shoulder finish line designs specifically in zirconia crown restorations. Therefore, further investigations are required to better understand the influence of these margin configurations on the marginal integrity of zirconia-based restorations.

## Conclusion:

We show marginal adaptation of CAD/CAM zirconia crowns was influenced by finish line design. The rounded shoulder finish line demonstrated superior marginal adaptation compared to the chamfer, although both remained within clinically acceptable limits (<120 µm). The shoulder bevel did not show any advantage over the sloping shoulder design, which may be preferred in anterior restorations due to improved esthetics and a biologically favorable margin.

## Advancement to knowledge:

This study provides comparative evidence on how different finish line designs influence the marginal adaptation of zirconium dioxide crowns under standardized *in vitro* conditions. The findings may help clinicians understand the effect of preparation design on marginal fit and assist in selecting appropriate finish lines for improved prosthetic outcomes.
